# Amplitude of Low-Frequency Fluctuations in Multiple-Frequency Bands in Acute Mild Traumatic Brain Injury

**DOI:** 10.3389/fnhum.2016.00027

**Published:** 2016-02-01

**Authors:** Jie Zhan, Lei Gao, Fuqing Zhou, Lijun Bai, Hongmei Kuang, Laichang He, Xianjun Zeng, Honghan Gong

**Affiliations:** ^1^Department of Radiology, The First Affiliated Hospital of Nanchang University, Nanchang, China; ^2^The Key Laboratory of Biomedical Information Engineering, Department of Biomedical Engineering, School of Life Science and Technology, Ministry of Education, Xi’an Jiaotong University, Xi’an, China

**Keywords:** resting-state fMRI, mild traumatic brain injury, acute, amplitude of low-frequency fluctuations, frequency band

## Abstract

Functional disconnectivity during the resting state has been observed in mild traumatic brain injury (mTBI) patients during the acute stage. However, it remains largely unknown whether the abnormalities are related to specific frequency bands of the low-frequency oscillations (LFO). Here, we used the amplitude of low-frequency fluctuations (ALFF) to examine the amplitudes of LFO in different frequency bands (slow-5: 0.01–0.027 Hz; slow-4: 0.027–0.073 Hz; and typical: 0.01–0.08 Hz) in patients with acute mTBI. A total of 24 acute mTBI patients and 24 age-, sex-, and education-matched healthy controls participated in this study. In the typical band, acute mTBI patients showed lower standardized ALFF in the right middle frontal gyrus and higher standardized ALFF in the right lingual/fusiform gyrus and left middle occipital gyrus. Further analyses showed that the difference between groups was concentrated in a narrower (slow-4) frequency band. In the slow-5 band, mTBI patients only exhibited higher standardized ALFF in the occipital areas. No significant correlation between the mini-mental state examination score and the standardized ALFF value was found in any brain region in the three frequency bands. Finally, no significant interaction between frequency bands and groups was found in any brain region. We concluded that the abnormality of spontaneous brain activity in acute mTBI patients existed in the frontal lobe as well as in distributed brain regions associated with integrative, sensory, and emotional roles, and the abnormal spontaneous neuronal activity in different brain regions could be better detected by the slow-4 band. These findings might contribute to a better understanding of local neural psychopathology of acute mTBI. Future studies should take the frequency bands into account when measuring intrinsic brain activity of mTBI patients.

## Introduction

Mild traumatic brain injury (mTBI) is a substantial neurological disorder that comprises approximately 80% of the 1.5 million traumatic brain injuries suffered each year in the United States (Nathan et al., 2015). It is characterized by subtle cognitive deficits within the first weeks after injury and typically resolves within 3–6 months post-injury (Walker and Tesco, 2013; Mayer et al., 2014, 2015). However, a subset of individuals (estimated to be 15%) develop post-concussion syndromes, leading to persistent neurological, cognitive, and behavioral symptoms, including headaches, memory problems, depression, and other features, that can impair social functioning and quality of life (Jeter et al., 2013; Walker and Tesco, 2013). Experimental injury models demonstrate that mTBI is capable of producing diffuse axonal injury. Unfortunately, conventional neuroimaging techniques (e.g., computed tomography or magnetic resonance imaging) do not have adequate sensitivity to diagnose and predict clinical outcomes of mTBI.

Recently, resting-state functional magnetic resonance imaging (RS-fMRI) has emerged as a promising tool for examining temporal correlations between low-frequency oscillations (LFO) in subjects with mTBI during the early stage (Johnson et al., 2012; Iraji et al., 2015; Mayer et al., 2015). LFO of the resting-state blood oxygen level-dependent (BOLD) signals is thought to reflect spontaneous neuronal activity of the brain (Biswal et al., 1995). Using a series of approaches, such as independent component analysis and seed-based linear correlation methods, several RS-fMRI studies have been conducted to examine the alterations of network connectivity in patients with mTBI (Johnson et al., 2012; Stevens et al., 2012; Sours et al., 2013; Iraji et al., 2015), supporting the idea that mTBI is linked to a disruption in functional connectivity within and between brain systems. Of note, most RS-fMRI studies that focused on the relationship between functional connectivity and clinical symptoms used acutely traumatized samples (Johnson et al., 2012; Shumskaya et al., 2012; Sours et al., 2013), as patients were imaged in acute time ranges of approximately 2 weeks after injury. Moreover, because it is at the acute stage that most mTBI patients report neurocognitive problems, detection of the neural basis of brain injury at the acute stage will be most likely to shed light on the link between early functional abnormalities and the possibility of protracted symptoms.

While functional connectivity can reveal the synchronization of LFO between spatially distinct brain regions, the regional properties of spontaneous brain activity in acute mTBI are less clear. The amplitude of low-frequency fluctuations (ALFF), which measures the total power of a given time course within a typical frequency range (e.g., 0.01–0.08 Hz), has been proven to be a valuable parameter to reflect the power of regional spontaneous neuronal activity (Zang et al., 2007). ALFF has been widely applied to evaluate LFO amplitudes of pathological brains, including schizophrenia (Hoptman et al., 2010), mild cognitive impairment (Bai et al., 2011; Han et al., 2012), Alzheimer’s disease (Wang et al., 2011), major depressive disorder (Wang et al., 2012), sleep-deprived brain (Gao et al., 2015), and traumatic brain injury (TBI) (Palacios et al., 2013; Zhou et al., 2014). The studies by Palacios et al. (2013) and Zhou et al. (2014), which suggested that the functional brain abnormalities of TBI can also be studied with ALFF, are of particular interest to the current study. Palacios et al. (2013) explored group differences in ALFF (0.01–0.08 Hz) between chronic and diffuse TBI patients (a mean of 4.1 years post-injury) and matched healthy volunteers, showing that patients with TBI had higher ALFF in the frontal regions, which was correlated with cognitive performance. Zhou et al. (2014) investigated the fractional ALFF (fALFF) in subacute mTBI patients (a mean of 23 days post-injury) in the range of 0.01–0.08 Hz. fALFF measures the power within a specific frequency range divided by the total power in the entire detectable frequency range (0–0.25 Hz) (Zou et al., 2008). Zhou et al. (2014) focused primarily on examining thalamic and cortical injuries in mTBI patients and found lower fALFF in the thalamus and the frontal and temporal lobes.

To date, it remains largely unknown whether mTBI patients show abnormal changes in LFO at the acute stage (<2 weeks post-injury). Recent studies (Zuo et al., 2010; Baria et al., 2011) have demonstrated that the oscillatory dynamics of the BOLD signal are sensitive to specific frequency bands. Buzsáki and Draguhn (2004) noted that brain neural oscillations cover a wide range of frequencies (0.05–500 Hz), including slow-5 (0.01–0.027 Hz), slow-4 (0.027–0.073 Hz), slow-3 (0.073–0.198 Hz), and slow-2 (0.198–0.25 Hz). The architecture of functional cortical networks in the brain appears to be related to systematic neural oscillations that occur in several oscillatory bands. Zuo et al. (2010) have shown that the ALFF in the slow-4 band (0.027–0.073 Hz) was higher than that in the slow-5 band (0.01–0.027 Hz) in a wide range of brain regions, such as the basal ganglia, thalamus, and precuneus, suggesting that the pattern of intrinsic brain activity is sensitive to specific frequency bands. Furthermore, it has been shown that patients with cognitive disorders exhibit frequency-dependent changes in abnormal LFO amplitudes (Hoptman et al., 2010; Han et al., 2012). Several other studies (Salvador et al., 2008; Baliki et al., 2011; Wee et al., 2012) also investigated the effects of different frequency bands on the global properties of whole-brain functional networks and brain states. Therefore, it would be necessary to differentiate the frequency bands to examine the LFO amplitudes in acute mTBI patients.

To address the above issues, we applied the ALFF approach to examine the amplitudes of LFO in acute mTBI patients at different frequency bands [slow-5 (0.01–0.027 Hz) and slow-4 (0.027–0.073 Hz) as well as the typical range (0.01–0.08 Hz)] to identify potential frequency-dependent changes. We sought to determine (i) whether acute mTBI patients show abnormal LFO amplitudes in brain regions that are vulnerable to damage in mTBI (such as the frontal lobe) based on prior neurophysiological investigations (Eierud et al., 2014) and (ii) whether the abnormalities are associated with specific frequency bands.

## Materials and Methods

### Subjects

From April 2013 to December 2014, 24 patients with acute mTBI [12 males; mean age, 39.0 ± 13.6 years (SD); educational attainment, 9.0 ± 3.5 years; mean time post-injury, 3.6 ± 3.3 days] and 24 sex-, age-, and education-matched (12 males; mean age, 40.2 ± 10.9 years; educational attainment, 8.9 ± 3.4 years) healthy controls (HC) participated in the study. All mTBI patients were recruited from the Department of Emergency, the First Affiliated Hospital of Nanchang University. HC were recruited from the local community by advertisements. The inclusion criteria for the mTBI group were as follows: (1) diagnosis of mTBI in the past 2 weeks and (2) age between 18 and 60 years. The exclusion criteria were as follows: (1) involvement in litigation; (2) presence or history of neurological and/or psychiatric conditions; or (3) history of substance or alcohol abuse. The mTBI diagnosis was made by a physician according to the following criteria (Borg et al., 2004): Glasgow Coma Scale (GCS) score of 13–15 (at first contact with medical staff) and the presence of one or more of the following manifestations: loss of consciousness limited to 30 min, post-traumatic amnesia limited to 24 h, and/or transient neurological abnormalities. None of the mTBI patients needed a neurosurgical intervention.

The mTBI subjects recruited for this study were an average of 3.6 ± 3.3 days post-injury with a range of 0.5–12 days post-injury. The injury mechanisms of mTBI patients included 15 motor vehicle accidents, 5 assaults, and 4 falls. Global cognitive performance was assessed using the mini-mental state examination (MMSE) (Folstein et al., 1975) within 24 h after an MRI scan examination. This study was approved by the Human Research Ethics Committee of the First Affiliated Hospital of Nanchang University, and informed written consent was obtained from all subjects.

### Data Acquisition

All images were collected on a 3.0-T (Siemens, Erlangen, Germany) scanner at the First Affiliated Hospital of Nanchang University. Foam pads were used to restrict head motion. Resting-state functional images were acquired using an echo-planar imaging (EPI) sequence: repetition time (TR) = 2,000 ms; echo time (TE) = 30 ms; flip angle = 90°; number of slices = 30; slice thickness = 4.0 mm; gap = 1.2 mm; field of view (FOV) = 200 mm × 200 mm; and matrix = 64 × 64.

During the RS-fMRI scanning, subjects were instructed to lie quietly in the scanner with their eyes closed. The fMRI scan lasted for 8 min and 6 s. However, the first 6 s was consumed by a dummy scan. Thus, we collected 240 volumes in total. In addition, we acquired high-resolution brain structural images for each subject by using a T1-weighted 3D MP-RAGE sequence (TR = 1,900 ms; TE = 2.26 ms; flip angle = 9°; FOV = 240 mm × 240 mm; matrix = 256 × 256; slice thickness = 1.0 mm; and 176 sagittal slices). Conventional T1- and T2-weighted images together with susceptibility-weighted images (SWI) were also collected on every participant to better characterize hemorrhagic or other lesions. The T1-, T2-, and SWI were carefully reviewed by two experienced radiologists (Xianjun Zeng and Laichang He), and lesions, if present, were documented.

### MRI Data Preprocessing

All preprocessing was performed using the Data Processing Assistant for Resting-State fMRI (DPARSF),^1^ which is based on Statistical Parametric Mapping (SPM8),^2^ and the Resting-State fMRI Data Analysis Toolkit (REST).^3^ For the resting-state fMRI data on each subject, the first 10 volumes were discarded to avoid the possible effects of scanner instability and adaptation of subjects to the circumstances. The remaining 230 volumes acquired from each subject were corrected for the differences in slice acquisition times. The resultant images were then realigned to correct for small movements that occurred between scans. Based on the recorded motion correction estimates, subjects with more than 2 mm maximum displacement in any of the *x*, *y*, or *z* directions or more than 2° of angular rotation about any axis for any of the 230 volumes were excluded from this study. Individual T1-weighted structural images were co-registered to the mean of the realigned EPI images. The transformed structural images were then segmented into gray matter, white matter, and cerebrospinal fluid (Ashburner and Friston, 2005). The Diffeomorphic Anatomical Registration Through Exponentiated Lie Algebra (DARTEL) tool (Ashburner, 2007) was used to compute the transformations from individual native space to MNI space. As RS-fMRI measures have been shown to be sensitive to micro-head motions (Yan et al., 2013a,b), the Friston 24-parameter model (Friston et al., 1996; Yan et al., 2013a) was used to regress head motion effects out of the realigned data (the 24 parameters include 6 head motion parameters, 6 head motion parameters one time point before, and the 12 corresponding squared items) based on recent reports that have demonstrated that higher order models benefit from the removal of head motion effects (Satterthwaite et al., 2013; Yan et al., 2013a,b). We further characterized the framewise displacement (FD), which considers measures of voxel-wise differences in motion in its derivation (Jenkinson et al., 2002;Power et al., 2012;Van Dijk et al., 2012;Yan et al., 2013a), as a measure of the micro-head motion of each subject (Yan et al., 2013a,b). Using the DPARSF toolbox, we computed the voxel-specific head motion, i.e., the values of mean voxel-specific FD for each subject. Group differences in the mean voxel-specific FD were calculated using a two-sample *t*-test, and the results were not statistically significant (*p* > 0.05) (for head motion parameters, see Table S1 in Supplementary Material). The mean voxel-specific FD was used as a covariate in the group comparisons of ALFF. To further reduce the effects of confounding factors, the signals from the white matter and cerebrospinal fluid, the mean time series of all voxels across the whole brain and linear and quadratic trends were removed from the data with linear regression (Yan et al., 2013a,b). The ALFF calculation was then performed.

### ALFF Calculation

For each subject, we calculated the ALFF value at each voxel. Specifically, the time series was first converted to the frequency domain using a Fast Fourier Transform, and the square root of the power spectrum was computed and then averaged across the predefined frequency interval. This averaged square root was termed ALFF, which measures the power of LFO.

To investigate alterations following acute mTBI, we calculated ALFF in the typical frequency band (0.01–0.08 Hz), slow-4 band (0.027–0.073 Hz), and slow-5 band (0.01–0.027 Hz). The voxel-wise ALFF map for each subject was then converted into a *z*-score map by subtracting the mean ALFF across the entire brain and dividing by the SD of the whole-brain ALFF (Zang et al., 2007).

### Statistical Analysis

Demographic and clinical variables were compared using the SPSS 17.0 software package (SPSS Inc., Chicago, IL, USA). Two-sample *t*-tests were performed to assess the differences in age, duration of education, and MMSE score between patients and HC. We set the significance level at *p* < 0.05.

For ALFF, we first conducted one-sample two-tailed *t*-tests to determine the within-group effects across the frequency bands (typical, slow-4, and slow-5), and these results were considered significant at a threshold of *p* < 0.05, corrected for false discovery rate (FDR). Then, a second-level independent two-sample *t*-test was performed to determine the difference between groups in the typical frequency band (0.01–0.08 Hz). To determine the main effects of frequency band, group, and their interactions, we performed a two-way analysis of variance (ANOVA) on a voxel-by-voxel basis with group (acute mTBI patients and HC) as a between-subject factor and frequency band (slow-4 and slow-5) as a repeated measures factor. We used Gaussian random field (GRF) correction, i.e., clusters with a voxel-level *p*-value <0.01 and cluster-level *p* < 0.05, to obtain a significant difference between the two groups. In the calculations, the confounding covariates, including age and gender, were controlled as covariates.

In addition, to seek evidence that altered spontaneous brain activity associated with the cognitive functioning, regional correlation analyses were conducted between the MMSE score and the cluster mean *z*-score of each patient within the mask of significant group differences. These correlations were also controlled for age, gender, and education. A *p*-value <0.05 was considered statistically significant.

## Results

### Demographic and Clinical Data

Table 1 shows the demographic and clinical data of all subjects. There were no significant differences in age and years of education between the acute mTBI patients and the HC. Acute mTBI patients showed significantly decreased MMSE score compared with HC (*p* = 0.003). Only 4/24 (16.7%) patients were diagnosed with scalp swelling on both the T2WI and SWI; the others had no abnormalities on the T2WI and SWI.

**Table 1:** **Demographic and clinical features of acute mTBI patients and HC**.

Characteristics	mTBI (*n* = 24)	HC (*n* = 24)	*p*-value
Gender (male/female)	12/12	12/12	>0.99^a^
Age (years)	39.0 ± 13.6	40.2 ± 10.9	0.718^b^
Education (years)	9.0 ± 3.5	8.9 ± 3.4	0.898^b^
GCS	14.4 ± 0.9		
MMSE	28.8 ± 1.1	29.5 ± 0.6	0.003^b^

*Data are presented as mean ± SD. mTBI, mild traumatic brain injury; HC, healthy controls; GCS, Glasgow Coma Scale; MMSE, mini-mental state examination*.

*^a^*p*-value was obtained using the two-tailed Chi-squared test*.

*^b^*p*-value was obtained by the two-sample *t*-test*.

### ALFF Analyses in Typical Frequency Band (0.01–0.08 Hz)

**Figure 1:**
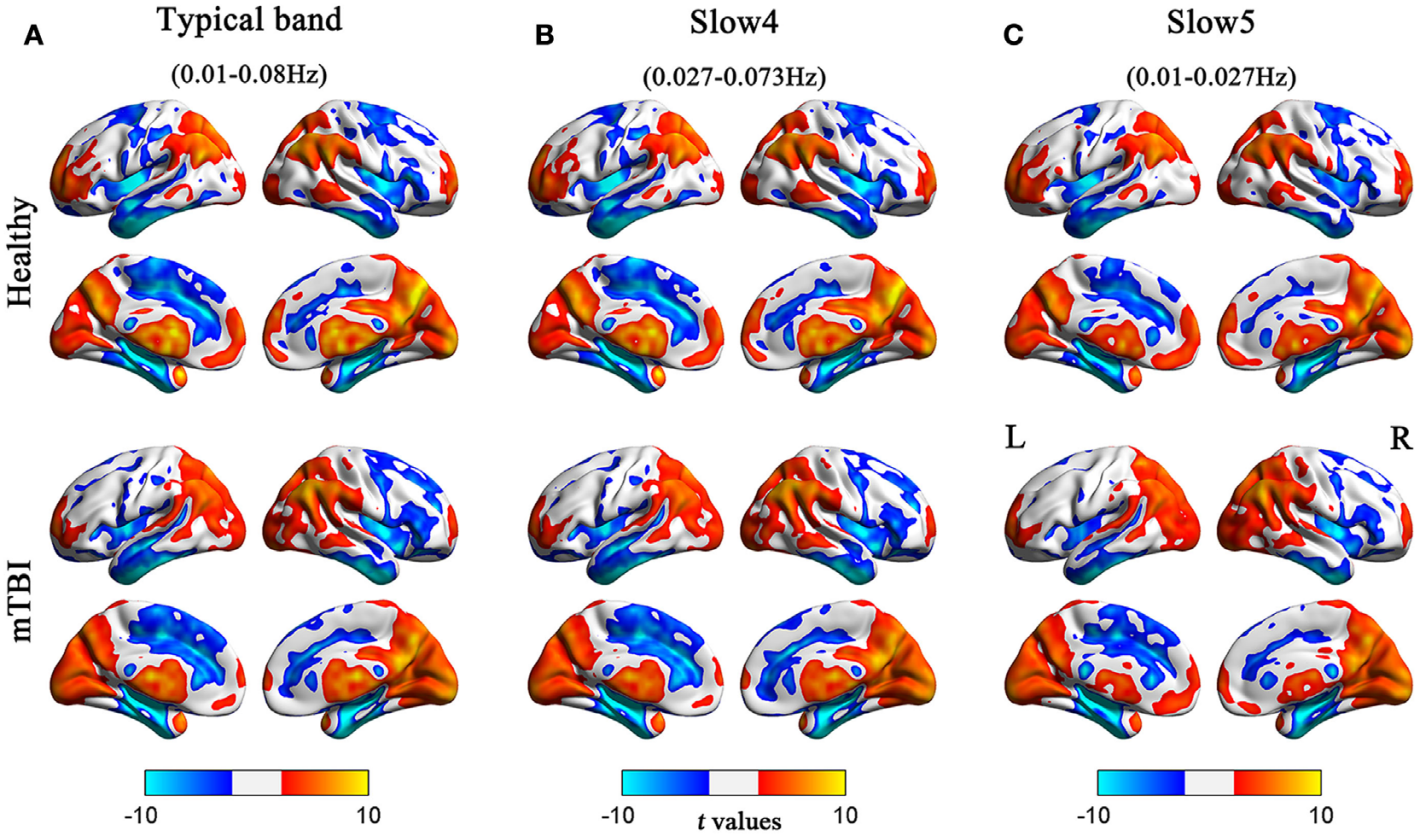
**Regions of significant ALFF in the HC and acute mTBI groups in the (A) typical frequency band (0.01–0.08 Hz), (B) slow-4 band (0.027–0.073 Hz), and (C) slow-5 band (0.01–0.027 Hz) separately**. The effects are significant at *p* < 0.05, FDR corrected; hot color indicates higher ALFF during resting state, and cool color indicates that the group had lower ALFF compared with its whole-brain mean. Left in the figure indicates the left side of the brain.

Before comparing the between-group ALFF differences, we first assessed the whole-brain ALFF results across the different frequency bands (typical, slow-4, and slow-5). For both the acute mTBI and HC groups, there were significantly higher standardized ALFF values than the global averaged values in some regions, including the visual cortex, posterior cingulate cortex (PCC)/precuneus, bilateral thalami, bilateral ventral medial prefrontal cortices (VMPFC), bilateral middle temporal gyri (MTG), and dorsolateral prefrontal cortex (DLPFC), mainly along the midline (see Figure 1).

**Figure 2:**
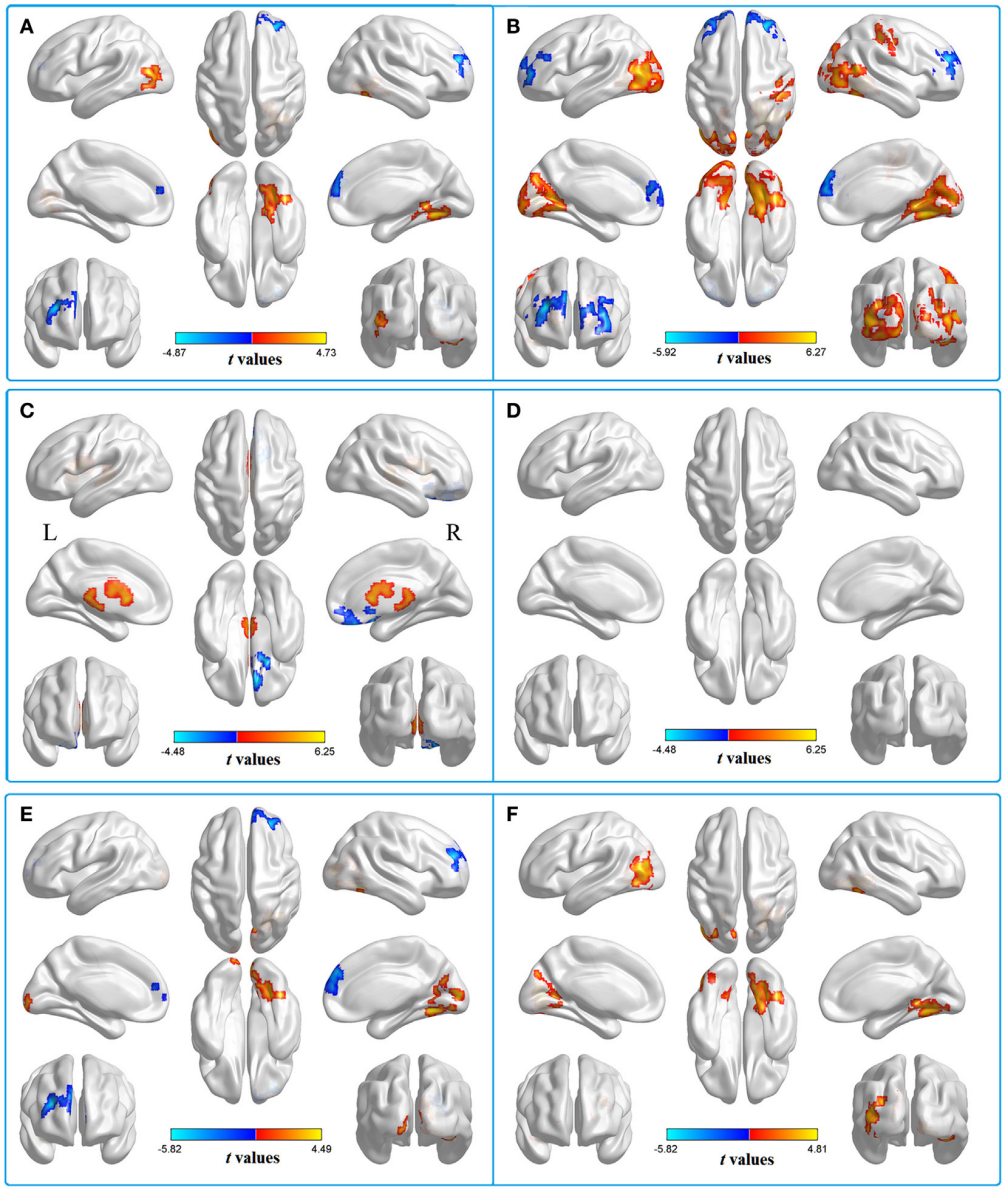
**Two-sample *t*-test for the (A) typical band (0.01–0.08 Hz) voxel-level *p* < 0.01 and cluster-level *p* < 0.05, GRF corrected**. Cool color indicates that the mTBI group had decreased ALFF compared with the controls and the hot color indicates the opposite. Main effects of **(B)** group. Hot color represents higher ALFF in the mTBI group than in the control group, whereas cool color represents lower ALFF in the mTBI group. Main effects of **(C)** frequency band. Hot color represents higher ALFF in the slow-4 band than in the slow-5 band, while cool color represents the opposite. The results were obtained by a 2 × 2 ANOVA. **(D)** Interaction between frequency bands and groups. Two-sample *t*-test for the **(E)** slow-4 band (0.027–0.073 Hz) and **(F)** slow-5 band (0.01–0.027 Hz), voxel-level *p* < 0.01 and cluster-level *p* < 0.05, GRF corrected. Cool color indicates that the mTBI group had decreased ALFF compared with the controls and the hot color indicates the opposite.

We then contrasted these ALFF patterns between the two groups, thereby identifying the inter-group differences in the typical frequency band. Compared with HC, patients with acute mTBI showed lower standardized ALFF in the right middle frontal gyrus [Brodmann’s area (BA) 10]. Patients also exhibited higher standardized ALFF in the right lingual/fusiform gyrus (BA 19/18/37) and left middle occipital gyrus (BA 19) (shown in Figure 2A; Table 2).

**Table 2:** **Comparisons of ALFF at typical frequency band (0.01–0.08 Hz) between groups**.

Brain regions	Brodmann’s area	MNI coordinates			*t*-value	Voxels
		*x*	*y*	*z*		
**HC** > **patients**						
R middle frontal gyrus	10	27	48	24	−4.8678	199
**Patients** > **HC**						
R lingual/fusiform gyrus	19/18/37	24	−57	−12	4.7302	253
L middle occipital gyrus	19	−36	78	15	4.0536	125

*MNI, Montreal Neurological Institute; *x*, *y*, *z*, coordinates of primary peak locations in the space of MNI; *t*, statistical value of peak voxel; L, left; R, right*.

*Comparisons were performed at voxel-level *p* < 0.01 and cluster-level *p* < 0.05, GRF corrected*.

### ALFF Changes in Different Frequency Bands

To test the frequency-specific changes in ALFF, we carried out a two-way 2 (groups) × 2 (frequency bands) ANOVA analysis. There was a main effect on group, and the significant differences were located in the bilateral middle frontal gyri (BA 10), left posterior cerebellum lobe (mTBI < HC), bilateral middle occipital gyri (BA 18/19), and right postcentral gyrus (BA 3/2), extending into the right precentral regions (BA 4) (mTBI > HC) (Figure 2B). The main effect of frequency was presented in the right inferior frontal gyrus (BA 11/47/10) (slow-4 < slow-5) and left white matter (slow-4 > slow-5) (Figure 2C). No significant interaction between frequency band and group was observed (Figure 2D).

Further analyses revealed that mTBI patients showed lower standardized ALFF in the right middle frontal gyrus and higher standardized ALFF in the right lingual/fusiform gyrus (BA 19/18/37) and the bilateral cuneus/lingual gyri (BA 31/18/17) in the slow-4 band (Figure 2E; Table 3) and only exhibited higher standardized ALFF in the right lingual gyrus (BA 19) and left middle occipital gyrus (BA 19/18) in the slow-5 band (Figure 2F; Table 4). Furthermore, to test whether the exclusion of four patients with scalp swelling would impact the current results, we repeated the analyses using 20 patients versus 20 controls, and we found that the between-group differences in ALFF across different frequency bands, main effects and the interactions were not substantially affected by the selection of patients. For the detailed results, please refer to Table S2 in Supplementary Material.

**Table 3:** **In the slow-4 band (0.027–0.073 Hz), group ALFF differences at the given threshold**.

Brain region	Brodmann’s area	MNI coordinates			*t*-value	Voxels
		*x*	*y*	*z*		
**HC** > **patients**						
R middle frontal gyrus	10/9	27	48	24	−5.8185	320
**Patients** > **HC**						
R lingual/fusiform gyrus	19/18/37	24	−60	−12	4.4909	165
L/R cuneus/lingual gyrus	31/18/17	15	−72	21	3.9899	169

*MNI, Montreal Neurological Institute; *x*, *y*, *z*, coordinates of primary peak locations in the space of MNI; *t*, statistical value of peak voxel; L, left; R, right*.

*Comparisons were performed at voxel-level *p* < 0.01 and cluster-level *p* < 0.05, GRF corrected*.

**Table 4:** **In the slow-5 band (0.01–0.027 Hz), group ALFF differences at the given threshold**.

Brain region	Brodmann’s area	MNI coordinates			*t*-value	Voxels
		*x*	*y*	*z*		
**Patients** > **HC**						
R lingual gyrus	19	36	−54	−12	4.5125	275
L middle occipital gyrus	19/18	−36	−78	15	4.8077	389

*MNI, Montreal Neurological Institute; *x*, *y*, *z*, coordinates of primary peak locations in the space of MNI; *t*, statistical value of peak voxel; L, left; R, right*.

*Comparisons were performed at voxel-level *p* < 0.01 and cluster-level *p* < 0.05, GRF corrected*.

Finally, no significant correlation between MMSE score and standardized ALFF value was found in any brain region in the three frequency bands.

## Discussion

We investigated the LFO amplitudes in patients with acute mTBI in different frequency bands (typical, slow-4, and slow-5) of the resting-state brain. We found that acute mTBI patients showed lower standardized ALFF in the right middle frontal gyrus and higher standardized ALFF in the right lingual/fusiform gyrus and left middle occipital gyrus. The abnormal spontaneous neuronal activity in different brain regions could be better detected by the slow-4 band. These findings might contribute to a better understanding of the pathophysiology of acute mTBI.

Although the origins, relation, and specific physiological functions of different frequency bands have not been fully clarified, neighboring bands were found to be typically associated with different brain states and competed with each other. The lowest-frequency band has the highest power and localizes mainly to the prefrontal, parietal, and occipital cortices; the higher-frequency band exhibits less power, and localizes mainly within the subcortical structures (e.g., thalamus and basal ganglia) (Baria et al., 2011). Different oscillatory bands are generated by different mechanisms and have different physiological functions. In our study, we found that the slow-4 band is more sensitive in detecting changes of spontaneous brain activity in the frontal regions. The mechanism of these changes is an interesting topic for future research.

In this study, it is important to note that the acute mTBI patients showed significant lower standardized ALFF in the right middle frontal gyrus in both the typical band and slow-4 band. The results were consistent with a previous study by Metting et al. (2009) that used perfusion CT imaging to measure changes in perfusion in acute mTBI patients and showed hypoperfusion in the frontal cortex. Additionally, a great number of studies using positron emission tomography (PET) have consistently reported frontal hypometabolism in mTBI patients at the acute and chronic stages (Gross et al., 1996; Bonne et al., 2003; Chen et al., 2003; Garcia-Panach et al., 2011; Byrnes et al., 2014). These studies showed a noticeable convergence of evidence demonstrating abnormalities in frontal lobe function after mTBI. Palacios et al. (2013) indicate that the decreased amplitude of LFO in neurodegenerative diseases probably reflects a loss of neurons that consecutively provoke connectivity deficits and disorganization or breakdown of brain networks. mTBI may damage the structure of neurofilaments and cause diffuse axonal injury, leading to microscopic lesions, myelin loss, and axonal degeneration or swelling, with axonal pathology being more pronounced in the acute phase of injury (Mac et al., 2007; Spain et al., 2010). Based on these findings, we speculated that the lower standardized ALFF of the right middle frontal gyrus probably reflected compromised frontal functioning in the acute mTBI patients. It is noteworthy that we also computed fALFF in our study. We found that the results of fALFF and ALFF analyses are similar. For the details of fALFF results, please refer to Table S3 in Supplementary Material.

We also observed higher standardized ALFF in acute mTBI patients, predominantly in the right lingual/fusiform gyrus and left middle occipital gyrus in the typical band; higher standardized ALFF in the right lingual/fusiform gyrus (BA 19/18/37) and bilateral cuneus/lingual gyri (BA 31/18/17) in the slow-4 band; and higher standardized ALFF in the right lingual gyrus (BA 19) and left middle occipital gyrus (BA 19/18) in the slow-5 band. In the current study, the potential mechanism for the increased spontaneous activity in the occipital regions, cuneus and fusiform gyrus is speculative, but previous studies have indicated that the visual association cortex and cuneus are responsible for visual associations and processing visual imagery, and the fusiform gyrus is a component of the ventral stream of the visual system (Mahon et al., 2007). Thus, we suggest that increased spontaneous neuronal activity in the occipital, cuneus, and fusiform areas in acute mTBI individuals at rest may indicate that patients were experiencing mental images of the trauma unconsciously in this resting condition.

In addition to the frontal and occipital lobes, brain regions with a significant main effect of group were identified in the right postcentral gyrus (BA 3/2), extending into the right precentral regions (BA 4) (mTBI > HC), and the left posterior cerebellum lobe (mTBI < HC) (Figure 2B). The postcentral and precentral gyri constitute the motor and sensory networks. Mazard et al. (2005) found that the sensorimotor areas were jointly activated with the occipital visual areas during mental imagery tasks. Previous RS-fMRI studies have also shown that the fluctuations in BOLD signals of the precentral and the postcentral gyri were highly correlated with those of the occipital visual areas (Nir et al., 2006; Wang et al., 2008). Therefore, the higher standardized ALFF in the postcentral and precentral regions could be explained by the increases of spontaneous neuronal activity in the visual cortex. The posterior cerebellum is predominantly involved in cognition regulation (Baillieux et al., 2010) and is recognized to be implicated in emotional modulation (Schmahmann and Caplan, 2006). Using ALFF, Sui et al. (2010) suggested that the cerebellum lobe was related to the neuropathology of cognitive and emotional processing of post-traumatic stress disorder (PTSD) patients. Yin et al. (2011) also showed that patients with PTSD had reduced spontaneous activity in the cerebellum. While concurrent diagnoses of mTBI and PTSD are difficult due to common symptoms and problems with self-report assessments (Capehart and Bass, 2012), there may still be relatively high rates of PTSD within the population of patients who have experienced mTBI (Hoge et al., 2008; Rosenfeld and Ford, 2010; Graner et al., 2013). In our study, reduced spontaneous cerebellar activity in acute mTBI patients may subserve cognitive and emotional impairment disorders.

### Limitations

Some limitations in our study are worth noting. First, because the sample size was relatively small, the results of the current study may not be representative of mTBI in general. Future studies could use a larger sample size to increase the reliability. Second, we could not observe dynamic ALFF changes in different progressions of mTBI due to the cross-sectional group data. In future studies, more attention need to be paid to longitudinal changes in neuronal activity.

## Conclusion

We concluded that the abnormality of spontaneous brain activity in acute mTBI patients existed in the frontal lobe as well as in distributed brain regions associated with integrative, sensory, and emotional roles, and the abnormal spontaneous neuronal activity in different brain regions could be better detected by the slow-4 band. These findings might contribute to a better understanding of the local neural psychopathology of acute mTBI. Future studies should take the frequency bands into account when measuring intrinsic brain activity of mTBI patients.

## Author Contributions

All authors listed, have made substantial, direct and intellectual contribution to the work, and approved it for publication.

## Conflict of Interest Statement

The authors declare that the research was conducted in the absence of any commercial or financial relationships that could be construed as a potential conflict of interest.

## Footnotes

^1^http://www.restfmri.net

^2^http://www.fil.ion.ucl.ac.uk/spm

^3^http://www.restfmri.net
