# Supplementary material for: Amplitude of Low-Frequency Fluctuations in Multiple-Frequency Bands in Acute Mild Traumatic Brain Injury
Source: Front Hum Neurosci. 2016 Feb 1;10:27. doi: 10.3389/fnhum.2016.00027 (PMC4740947; doi:10.3389/fnhum.2016.00027)
Supplement: Supplementary file 1 [file Data_Sheet_1.DOC]

| **Table S1│Head motion parameters.** | | | | | |
| --- | --- | --- | --- | --- | --- |
| **Head Motion Parameters** | **mTBI**  **Mean ± SD** | **HC**  **Mean ± SD** | ***t*** | ***p-value*** | ***df*** |
| mean RMS | 0.3756 ± 0.22896 | 0.4321 ± 0.31162 | -0.894 | 0.376 | 46 |
| mean FD_Jenkinson | 0.0951 ± 0.07199 | 0.1016 ± 0.06360 | -0.210 | 0.835 | 46 |
| mean relative RMS  (mean FD_VanDijk) | 0.0426 ± 0.03360 | 0.0498 ± 0.04163 | -0.572 | 0.570 | 46 |
| mean FD_Power | 0.1763 ± 0.12955 | 0.1869 ± 0.10923 | -0.166 | 0.869 | 46 |
| Notes: RMS, root mean square; FD, framewise displacement; mean FD_Jenkinson was used due to its consideration of voxel-wise differences in motion in its derivation (Jenkinson et al., 2002); and see (Van Dijk et al., 2012) for mean FD_VanDijk; FD_Power is defined as the volume-based framewise displacement proposed in (Power et al., 2012). | | | | | |

**Reference**

Jenkinson M, Bannister P, Brady M, Smith S (2002) Improved optimization for the robust and accurate linear registration and motion correction of brain images. Neuroimage 17(2):825-841.

Van Dijk, KR, Sabuncu, MR, Buckner, RL (2012) The influence of head motion on intrinsic functional connectivity MRI. Neuroimage 59(1): 431-438.

# [Power JD](http://www.ncbi.nlm.nih.gov/pubmed/?term=Power JD%5BAuthor%5D&cauthor=true&cauthor_uid=22019881), [Barnes KA](http://www.ncbi.nlm.nih.gov/pubmed/?term=Barnes KA%5BAuthor%5D&cauthor=true&cauthor_uid=22019881), [Snyder AZ](http://www.ncbi.nlm.nih.gov/pubmed/?term=Snyder AZ%5BAuthor%5D&cauthor=true&cauthor_uid=22019881), [Schlaggar BL](http://www.ncbi.nlm.nih.gov/pubmed/?term=Schlaggar BL%5BAuthor%5D&cauthor=true&cauthor_uid=22019881), [Petersen SE](http://www.ncbi.nlm.nih.gov/pubmed/?term=Petersen SE%5BAuthor%5D&cauthor=true&cauthor_uid=22019881) (2012) Spurious but systematic correlations in functional connectivity MRI networks arise from subject motion. [Neuroimage](http://www.ncbi.nlm.nih.gov/pubmed/22019881) 59(3): 2142-2154.
