# Supplementary material for: Amplitude of Low-Frequency Fluctuations in Multiple-Frequency Bands in Acute Mild Traumatic Brain Injury
Source: Front Hum Neurosci. 2016 Feb 1;10:27. doi: 10.3389/fnhum.2016.00027 (PMC4740947; doi:10.3389/fnhum.2016.00027)
Supplement: Supplementary file 2 [file Data_Sheet_2.DOC]

**ALFF results of acute mTBI patients without scalp swelling**

| **Table S2A | Demographic and clinical features of acute mTBI patients (without scalp swelling) and HC.** | | | | | | | | | |
| --- | --- | --- | --- | --- | --- | --- | --- | --- | --- |
| **Characteristics** |  |  | **mTBI (n=20)** |  |  | **HC (n=20)** |  |  | ***p***-value |
| Gender (male/female) |  |  | 11/9 |  |  | 11/9 |  |  | >0.05^a^ |
| Age (years) |  |  | 39.4 ± 13.5 |  |  | 40.9 ± 11.1 |  |  | 0.704^b^ |
| Education (years) |  |  | 8.8 ± 3.8 |  |  | 8.8 ± 3.4 |  |  | 0.965^b^ |
| GCS |  |  | 14.5 ± 0.8 |  |  |  |  |  |  |
| MMSE |  |  | 28.7 ± 1.1 |  |  | 29.5 ± 0.6 |  |  | 0.005^b^ |
| Data are presented as mean ± SD. Abbreviations: mTBI, mild Traumatic Brain Injury; HC, Healthy Controls; GCS, Glasgow Coma Scale; MMSE, Mini-Mental State Examination. a, *p*-value was obtained using the two-tailed Chi-squared test. b, *p-*value was obtained by the two-sample t-test. | | | | | | | | | |

| **Table S2B | Comparisons of ALFF at typical frequency band (0.01**–**0.08Hz) between groups.** | | | | | | | |
| --- | --- | --- | --- | --- | --- | --- | --- |
| **Brain regions** |  | **Brodmann** | **MNI coordinates** | | | ***t*** value | **Voxels** |
|  |  | **area** | **x** | **y** | **z** |  |  |
| **HC > patients** |  |  |  |  |  |  |  |
| R Middle Frontal Gyrus |  | 10 | 30 | 51 | 21 | -4.7064 | 173 |
| **Patients > HC** |  |  |  |  |  |  |  |
| R Lingual/Fusiform Gyrus |  | 19/18/37 | 24 | -63 | -12 | 5.5877 | 580 |
| L Middle Occipital Gyrus |  | 19 | -45 | -72 | 9 | 4.4118 | 207 |
| L Cuneus/Lingual Gyrus |  | 18/31 | -9 | -78 | 27 | 4.0074 | 124 |
| MNI, Montreal Neurological Institute; x, y, z, coordinates of primary peak locations in the space of MNI; *t*, statistical value of peak voxel; L, left; R, right. Comparisons were performed at voxel-level *p* < 0.01 and cluster-level *p* < 0.05, GRF corrected. | | | | | | | |

| **Table S2C | In the slow-4 band (0.027–0.073Hz), group ALFF differences at the given threshold.** | | | | | | | |
| --- | --- | --- | --- | --- | --- | --- | --- |
| **Brain region** | | **Brodmann** | **MNI coordinates** | | | ***t* value** | **Voxels** |
|  |  | **area** | **x** | **y** | **z** |  |  |
| **HC > patients** |  |  |  |  |  |  |  |
| R Middle Frontal Gyrus |  | 10/9 | 27 | 48 | 24 | -5.3245 | 251 |
| **Patients > HC** |  |  |  |  |  |  |  |
| R Lingual/Fusiform Gyrus |  | 19/18/37 | 24 | -63 | -12 | 5.4251 | 606 |
| L Middle Temporal Gyrus |  | 39 | -48 | -72 | 9 | 4.2390 | 141 |
| L Calcarine |  | 18/31/19 | -24 | -60 | 9 | 3.9487 | 121 |
| MNI, Montreal Neurological Institute; x, y, z, coordinates of primary peak locations in the space of MNI; *t*, statistical value of peak voxel; L, left; R, right. Comparisons were performed at voxel-level *p* < 0.01 and cluster-level *p* < 0.05, GRF corrected. | | | | | | | |

| **Table S2D | In the slow-5 band (0.01**–**0.027 Hz), group ALFF differences at the given threshold.** | | | | | | | |
| --- | --- | --- | --- | --- | --- | --- | --- |
| **Brain region** | | **Brodmann** | **MNI coordinates** | | | ***t* value** | **Voxels** |
|  |  | **area** | **x** | **y** | **z** |  |  |
| **HC > patients** |  |  |  |  |  |  |  |
| L Cerebelum Posterior Lobe |  |  | -12 | -48 | -51 | -6.3137 | 168 |
| **Patients > HC** |  |  |  |  |  |  |  |
| R Middle Occipital Gyrus |  | 19/18 | 54 | -60 | 12 | 4.5740 | 176 |
| L Middle Occipital Gyrus |  | 19/18 | -36 | -78 | 15 | 5.2066 | 1185 |
| MNI, Montreal Neurological Institute; x, y, z, coordinates of primary peak locations in the space of MNI; *t*, statistical value of peak voxel; L, left; R, right. Comparisons were performed at voxel-level *p* < 0.01 and cluster-level *p* < 0.05, GRF corrected. | | | | | | | |

| **Table S2E | The main effects for group on ALFF.** | | | | | | | |
| --- | --- | --- | --- | --- | --- | --- | --- |
| **Brain region** | | **Brodmann** | **MNI coordinates** | | | ***t* value** | **Voxels** |
|  |  | **area** | **x** | **y** | **z** |  |  |
| **HC > patients** |  |  |  |  |  |  |  |
| L Middle Frontal Gyrus |  | 10 | -36 | 57 | 3 | -4.5146 | 199 |
| R Middle Frontal Gyrus |  | 10 | 30 | 51 | 21 | -5.9452 | 474 |
| L Cerebellum Posterior Lobe | |  | -12 | -45 | -51 | -6.2544 | 881 |
| **Patients > HC** |  |  |  |  |  |  |  |
| R Middle Occipital Gyrus |  | 18/19 | 24 | -63 | -12 | 7.3114 | 3455 |
| R Postcentral/Precentral Gyrus |  | 3/4/2 | 48 | -30 | 48 | 5.2101 | 233 |
| MNI, Montreal Neurological Institute; x, y, z, coordinates of primary peak locations in the space of MNI; *t*, statistical value of peak voxel; L, left; R, right. Comparisons were performed at voxel level *p* < 0.01 and cluster level *p* < 0.05, GRF corrected. | | | | | | | |

| **Table S2F | The main effects for frequency on ALFF.** | | | | | | | |
| --- | --- | --- | --- | --- | --- | --- | --- |
| **Brain region** | | **Brodmann** | **MNI coordinates** | | | ***t* value** | **Voxels** |
|  |  | **area** | **x** | **y** | **z** |  |  |
| **Slow-4 > Slow-5** |  |  |  |  |  |  |  |
| White matter |  |  | -3 | -39 | -30 | 6.3251 | 2071 |
| **Slow-5 > Slow-4** |  |  |  |  |  |  |  |
| R Inferior Frontal Gyrus |  | 47/11/25 | -21 | 12 | -21 | -3.5343 | 133 |
| R Medial Frontal Gyrus |  | 11/10 | 6 | 33 | -24 | -3.7141 | 112 |
| MNI, Montreal Neurological Institute; x, y, z, coordinates of primary peak locations in the space of MNI; *t*, statistical value of peak voxel; R, right. Comparisons were performed at voxel level *p* < 0.01 and cluster level *p* < 0.05, GRF corrected. | | | | | | | |
