# Supplementary material for: Amplitude of Low-Frequency Fluctuations in Multiple-Frequency Bands in Acute Mild Traumatic Brain Injury
Source: Front Hum Neurosci. 2016 Feb 1;10:27. doi: 10.3389/fnhum.2016.00027 (PMC4740947; doi:10.3389/fnhum.2016.00027)
Supplement: Supplementary file 3 [file Data_Sheet_3.DOC]

**fALFF Results**

| **Table S3A | Comparisons of fALFF at typical frequency band (0.01**–**0.08Hz) between groups.** | | | | | | | |
| --- | --- | --- | --- | --- | --- | --- | --- |
| **Brain regions** |  | **Brodmann** | **MNI coordinates** | | | ***t*** value | **Voxels** |
|  |  | **area** | **x** | **y** | **z** |  |  |
| **HC > patients** |  |  |  |  |  |  |  |
| L Precuneus Gyrus |  | 7/31 | 0 | -54 | 39 | -4.2753 | 129 |
| **Patients > HC** |  |  |  |  |  |  |  |
| R Lingual/Fusiform Gyrus |  | 19/18/37 | 27 | -42 | -3 | 4.2657 | 177 |
| L Middle Occipital Gyrus |  | 19 | -36 | -69 | -6 | 4.7261 | 371 |
| MNI, Montreal Neurological Institute; x, y, z, coordinates of primary peak locations in the space of MNI; *t*, statistical value of peak voxel; L, left; R, right. Comparisons were performed at voxel level *p* < 0.01 and cluster level *p* < 0.05, GRF corrected. | | | | | | | |

| **Table S3B | In the slow-4 band (0.027**–**0.073Hz), group fALFF differences at the given threshold.** | | | | | | | |
| --- | --- | --- | --- | --- | --- | --- | --- |
| **Brain region** | | **Brodmann** | **MNI coordinates** | | | ***t* value** | **Voxels** |
|  |  | **area** | **x** | **y** | **z** |  |  |
| **HC > patients** |  |  |  |  |  |  |  |
| R Medial Frontal Gyrus |  | 10 | 6 | 45 | -12 | -4.1461 | 147 |
| R Middle Frontal Gyrus |  | 10 | 6 | 60 | 27 | -3.8237 | 122 |
| L Precuneus |  | 7 | -3 | -51 | 36 | -3.8294 | 84 |
| **Patients > HC** |  |  |  |  |  |  |  |
| R Lingual/Fusiform Gyrus |  | 19/18/37 | 24 | -75 | -21 | 4.0509 | 285 |
| R Lingual/Fusiform Gyrus |  | 19/18/37 | 39 | -57 | -9 | 3.9985 | 83 |
| L Middle Occipital Gyrus |  | 19/18 | -30 | -51 | -27 | 5.1986 | 350 |
| MNI, Montreal Neurological Institute; x, y, z, coordinates of primary peak locations in the space of MNI; *t*, statistical value of peak voxel; L, left; R, right. Comparisons were performed at voxel level *p* < 0.01 and cluster level *p* < 0.05, GRF corrected. | | | | | | | |

| **Table S3C | In the slow-5 band (0.01**–**0.027 Hz), group fALFF differences at the given** | | | | | | | |
| --- | --- | --- | --- | --- | --- | --- | --- |
| **threshold.** | | | | | | | |
| **Brain region** | | **Brodmann** | **MNI coordinates** | | | ***t* value** | **Voxels** |
|  |  | **area** | **x** | **y** | **z** |  |  |
| **Patients > HC** |  |  |  |  |  |  |  |
| R Lingual/Fusiform Gyrus |  | 19/18/37 | 45 | -51 | -18 | 4.0103 | 129 |
| L Middle Occipital Gyrus |  | 19 | -45 | -69 | 9 | 4.8210 | 507 |
| MNI, Montreal Neurological Institute; x, y, z, coordinates of primary peak locations in the space of MNI; *t*, statistical value of peak voxel; L, left; R, right. Comparisons were performed at voxel level *p* < 0.01 and cluster level *p* < 0.05, GRF corrected. | | | | | | | |

| **Table S3D | The main effects for group on fALFF.** | | | | | | | |
| --- | --- | --- | --- | --- | --- | --- | --- |
| **Brain region** | | **Brodmann** | **MNI coordinates** | | | ***t* value** | **Voxels** |
|  |  | **area** | **x** | **y** | **z** |  |  |
| **HC > patients** |  |  |  |  |  |  |  |
| R Medial Frontal Gyrus |  | 10 | 21 | 63 | 30 | -4.3206 | 125 |
| L Middle Temporal Gyrus |  | 38 | -33 | 15 | -30 | -4.3390 | 136 |
| **Patients > HC** |  |  |  |  |  |  |  |
| R Middle Occipital Gyrus |  | 19 | 27 | -45 | -3 | 5.2949 | 818 |
| L Middle Occipital Gyrus |  | 18/19 | -42 | -66 | 15 | 4.9703 | 1083 |
| MNI, Montreal Neurological Institute; x, y, z, coordinates of primary peak locations in the space of MNI; *t*, statistical value of peak voxel; L, left; R, right. Comparisons were performed at voxel level *p* < 0.01 and cluster level *p* < 0.05, GRF corrected. | | | | | | | |

| **Table S3E | The main effects for frequency on fALFF.** | | | | | | | |
| --- | --- | --- | --- | --- | --- | --- | --- |
| **Brain region** | | **Brodmann** | **MNI coordinates** | | | ***t* value** | **Voxels** |
|  |  | **area** | **x** | **y** | **z** |  |  |
| **Slow-4 > Slow-5** |  |  |  |  |  |  |  |
| White matter |  |  | 33 | -45 | 27 | 10.3141 | 11671 |
| **Slow-5 > Slow-4** |  |  |  |  |  |  |  |
| R Lingual/Fusiform Gyrus |  | 19/18/37 | 12 | -63 | -57 | -5.0716 | 1357 |
| MNI, Montreal Neurological Institute; x, y, z, coordinates of primary peak locations in the space of MNI; *t*, statistical value of peak voxel; R, right. Comparisons were performed at voxel level *p* < 0.01 and cluster level *p* < 0.05, GRF corrected. | | | | | | | |
